# Supplementary material for: Analysis of Microbial Diversity and Metabolites in Sauerkraut Products with and without Microorganism Addition
Source: Foods. 2023 Mar 9;12(6):1164. doi: 10.3390/foods12061164 (PMC10048197; doi:10.3390/foods12061164)
Supplement: Supplementary file 1 [file foods-12-01164-s001.zip › Hightlights(1).pdf]

Hightlights

Different modes of microbial addition formed their own unique microbial communities;

Different strains have similar effects to inhibit *Firmicutes* production;

Fermented sauerkraut without microbes added was richer in amino acids and organic acids;

Alcohols, esters and acids were more abundant in fermented sauerkraut with microbes added;

Cystine, ascorbic acid and alcohols were closely associated with a wide range of microorganisms in sauerkraut.
