# Supplementary material for: Analysis of Microbial Diversity and Metabolites in Sauerkraut Products with and without Microorganism Addition
Source: Foods. 2023 Mar 9;12(6):1164. doi: 10.3390/foods12061164 (PMC10048197; doi:10.3390/foods12061164)
Supplement: Supplementary file 1 [file foods-12-01164-s001.zip › Supplementary Information Figure.pdf]

Figure S1. Microbial diversity analysis of sauerkraut. (A) weighted UniFrac distance, (B) unweighted UniFrac distance, (C) principal coordinates analysis.

Figure S2. Targeted metabolome analysis. (A) Amino acid score plot, (B) organic acid score plot.

Figure S3. Analysis of volatile compounds. (A) 2D drawings, (B) differential models, (C) VOC biplot scores.

Figure S4. Correlations between bacterial taxa and metabolites. (A) Network diagram of amino acids and bacterial taxa, (B) Network diagram of organic acids and bacterial taxa, (C) Network diagram of VOCs and bacterial taxa.

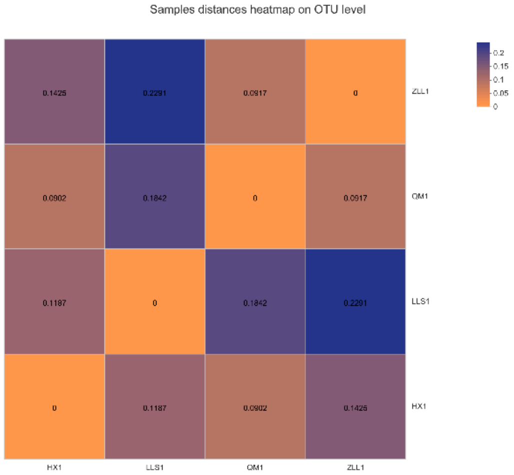

, S1A

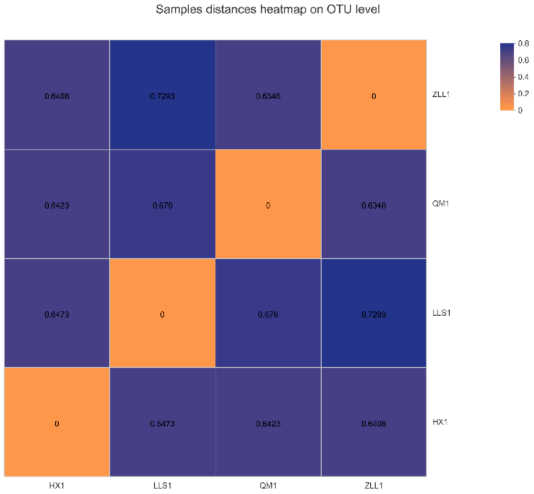

S1B

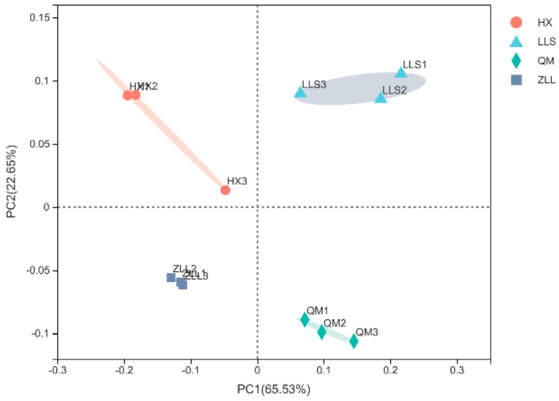
HX
LLS
QM
ZLL
PC2(22.65%)
PC1(65.53%)
HX1
HX2
HX3
LLS1
LLS2
LLS3
QM1
QM2
QM3
ZLL2
ZLL3
0.15
0.1
0.05
0
-0.05
-0.1
-0.3
-0.2
-0.1
0
0.1
0.2
0.3

S1C

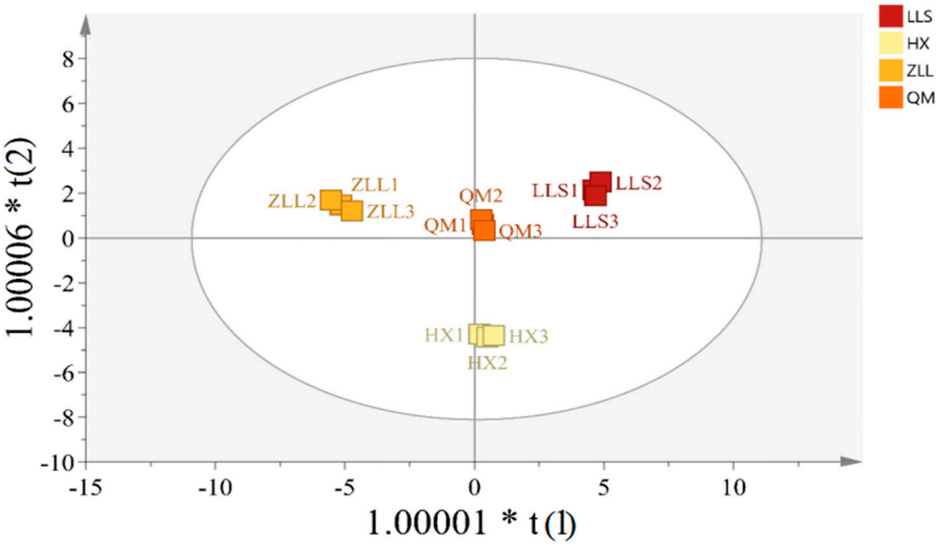
LLS
HX
ZLL
QM
1.00006 * t(2)
1.00001 * t(1)
ZLL1
ZLL2
ZLL3
QM1
QM2
QM3
LLS1
LLS2
LLS3
HX1
HX2
HX3
8
6
4
2
0
-2
-4
-6
-8
-10
-15
-10
-5
0
5
10

S2A

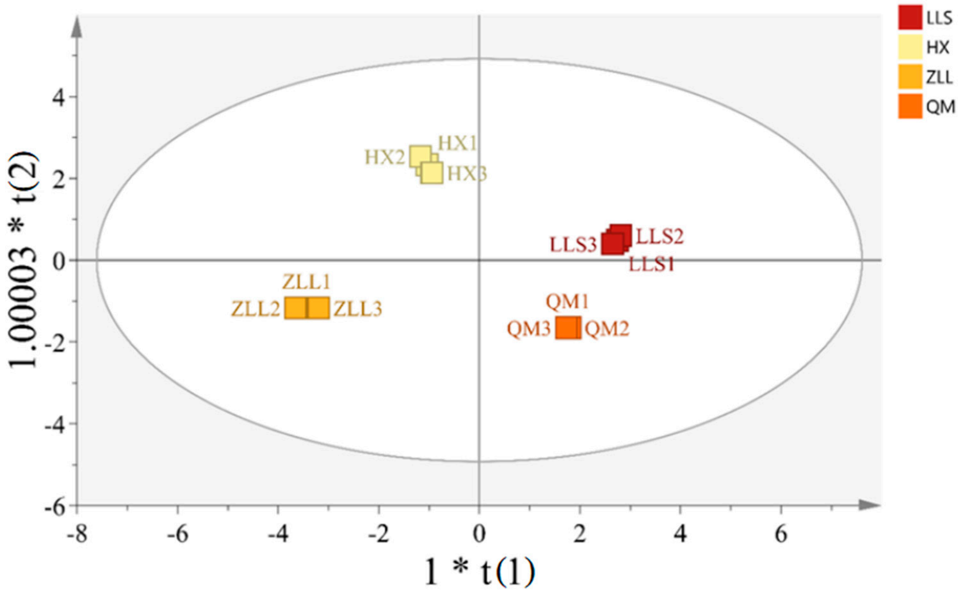
LLS
HX
ZLL
QM
1.00003 * t(2)
1 * t(1)
HX1
HX2
HX3
LLS1
LLS2
LLS3
ZLL1
ZLL2
ZLL3
QM1
QM2
QM3
4
2
0
-2
-4
-6
-8
-6
-4
-2
0
2
4
6

S2B

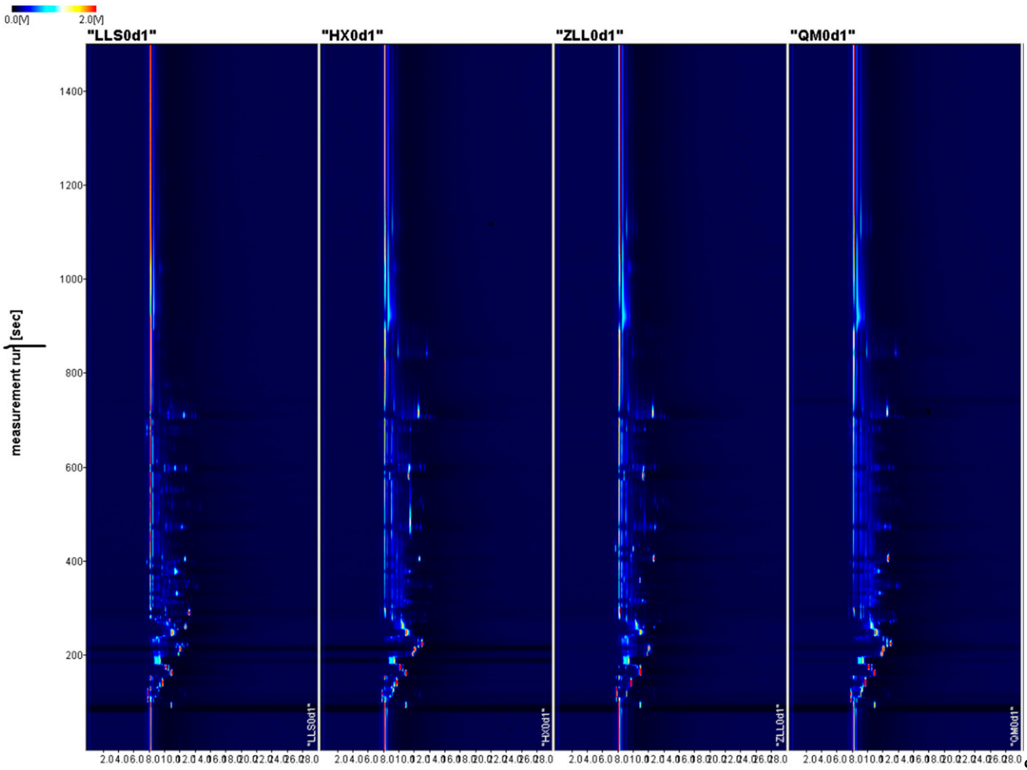
0.0[V]
2.0[V]
"LLS0d1"
"HX0d1"
"ZLL0d1"
"QM0d1"
measurement run [sec]

S3A

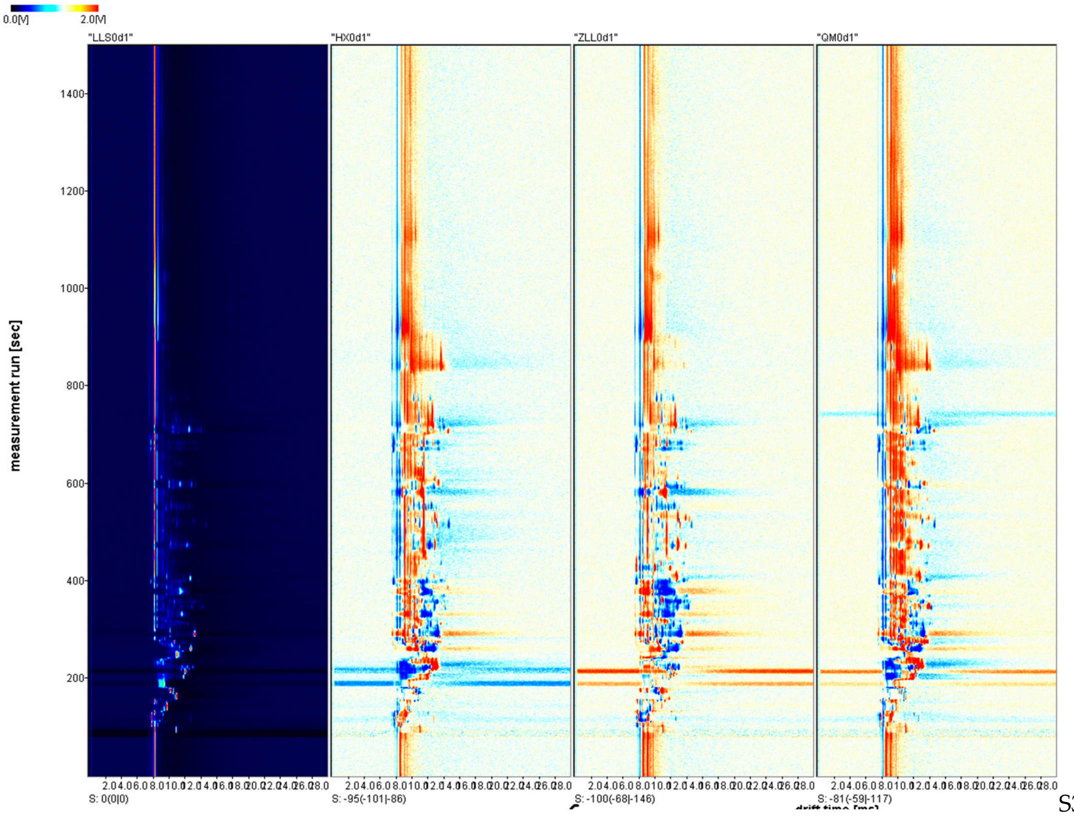
0.0[V]
2.0[V]
"LLS0d1"
"HX0d1"
"ZLL0d1"
"QM0d1"
measurement run [sec]
S: 0(0|0)
S: -95(-101|-86)
S: -100(-68|-146)
S: -81(-59|-117)

S3B

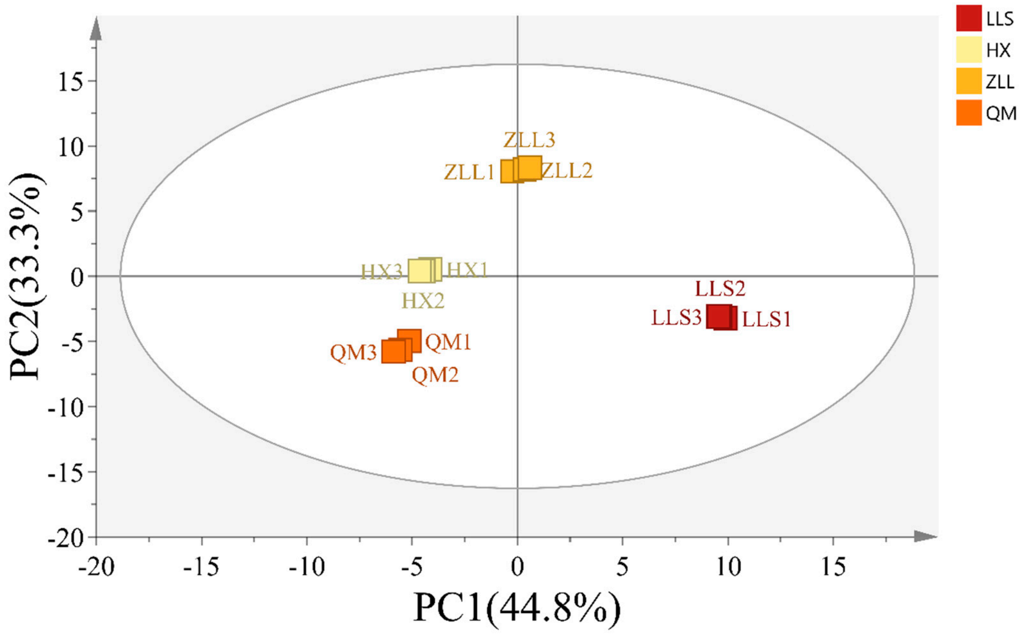

LLS
HX
ZLL
QM
ZLL3
ZLL1
ZLL2
HX3
HX1
HX2
LLS2
LLS3
LLS1
QM1
QM3
QM2
PC2(33.3%)
PC1(44.8%)
15
10
5
0
-5
-10
-15
-20
-20
-15
-10
-5
0
5
10
15

S3C

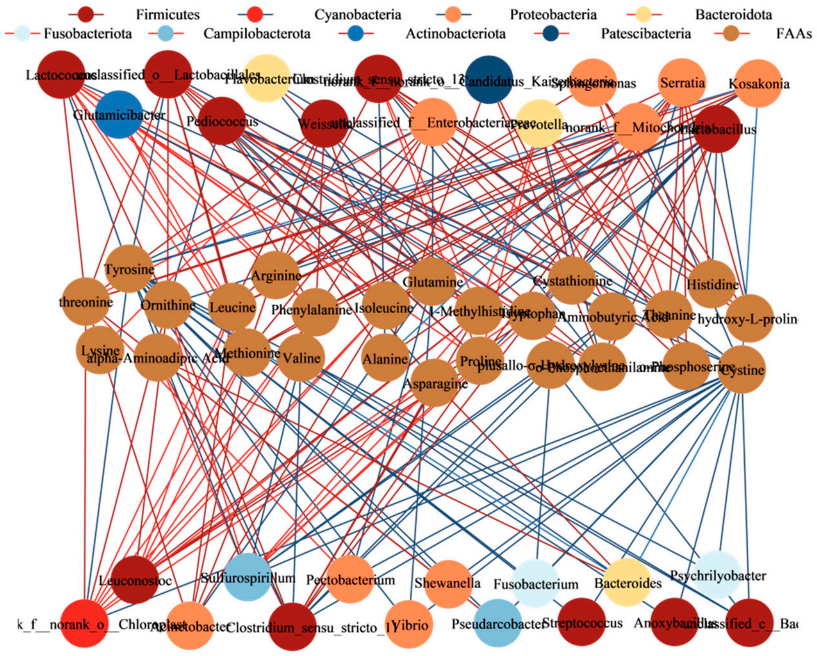

Firmicutes
Cyanobacteria
Proteobacteria
Bacteroidota
Fusobacteriota
Campilobacterota
Actinobacteriota
Patescibacteria
FAAs
Glutamicibacter
Pediococcus
Serratia
Kosakonia
Tyrosine
Arginine
Glutamine
Cystathionine
Histidine
threonine
Ornithine
Leucine
Phenylalanine
Isoleucine
Lysine
alpha-Aminoadipic Acid
Methionine
Valine
Alanine
Asparagine
Leuconostoc
Sulfurospirillum
Pectobacterium
Shewanella
Fusobacterium
Bacteroides
Psychrilyobacter
Vibrio
Streptococcus

S4A

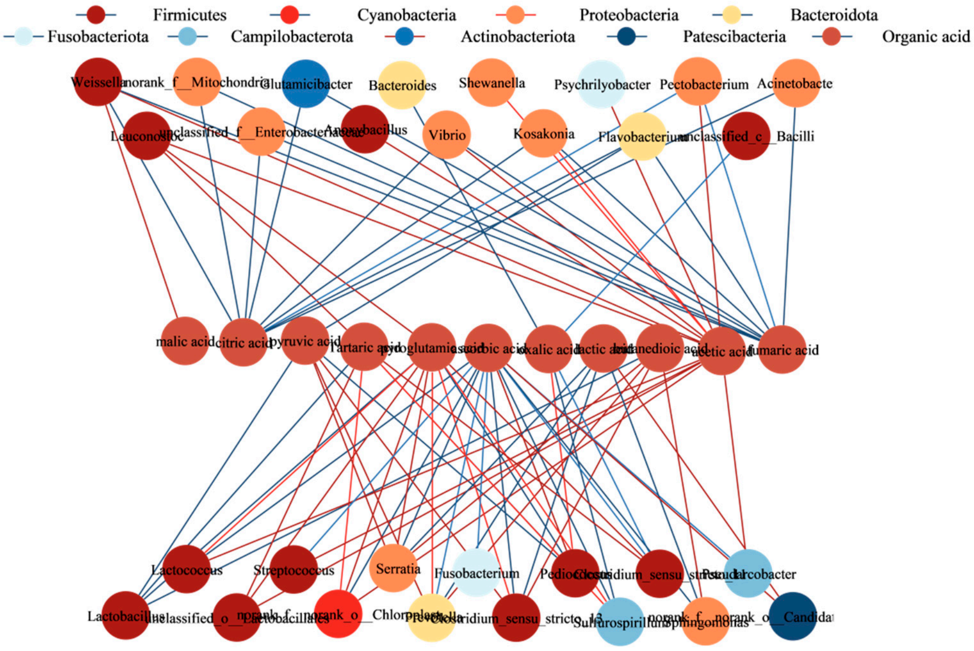
Firmicutes
Cyanobacteria
Proteobacteria
Bacteroidota
Fusobacteriota
Campilobacterota
Actinobacteriota
Patescibacteria
Organic acid
Weissella
Glutamicibacter
Bacteroides
Shewanella
Psychrilyobacter
Pectobacterium
Vibrio
Kosakonia
malic acid
citric acid
pyruvic acid
fumaric acid
Lactococcus
Streptococcus
Serratia
Fusobacterium
Lactobacillus

S4B

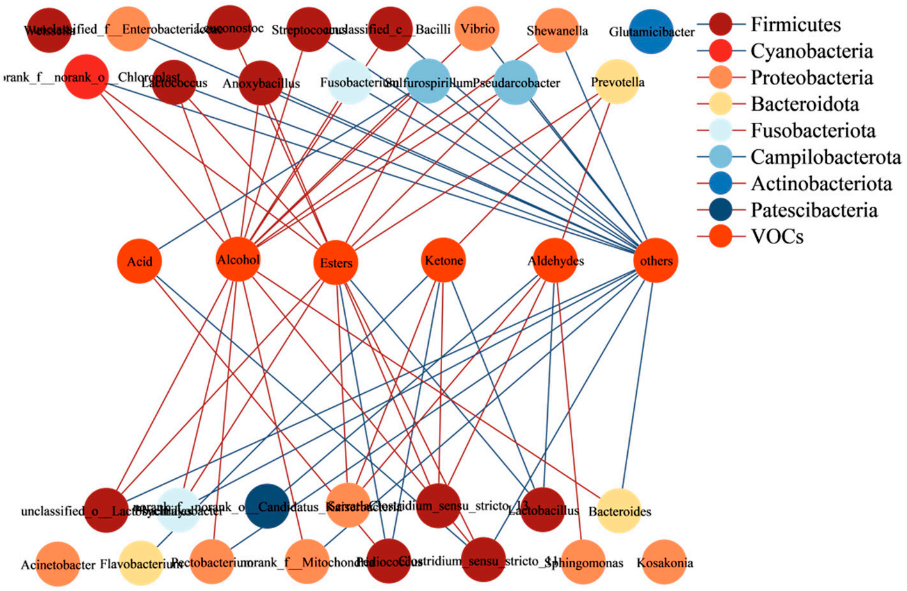
Firmicutes
Cyanobacteria
Proteobacteria
Bacteroidota
Fusobacteriota
Campilobacterota
Actinobacteriota
Patescibacteria
VOCs
Vibrio
Shewanella
Glutamicibacter
Prevotella
Anoxybacillus
Acid
Alcohol
Esters
Ketone
Aldehydes
others
Bacteroides
Acinetobacter
Sphingomonas
Kosakonia

S4C
