# Supplementary material for: Analysis of Microbial Diversity and Metabolites in Sauerkraut Products with and without Microorganism Addition
Source: Foods. 2023 Mar 9;12(6):1164. doi: 10.3390/foods12061164 (PMC10048197; doi:10.3390/foods12061164)
Supplement: Supplementary file 1 [file foods-12-01164-s001.zip › Supplementary Information Table.pdf]

Table S1. Relative amino acid contents.

Table S2. Relative contents of organic acids.

Table S3. List of compounds in sauerkraut.

Table S1

**Amino acid**	**LLS**	**HX**	**ZLL**	**QM**
Aminobutyric acid	20.81%	5.70%	3.07%	13.15%
Phenylalanine	16.84%	10.69%	18.00%	18.11%
Isoleucine	15.03%	11.29%	13.42%	18.44%
Leucine	15.03%	11.29%	13.42%	18.44%
Tryptophan	13.45%	24.04%	11.41%	12.76%
Valine	4.51%	1.06%	1.18%	0.85%
o-Phosphoethanilamine	3.07%	1.32%	0.64%	3.83%
Cystine	2.28%	0.78%	0.31%	0.43%
Lysine	1.88%	0.00%	1.69%	0.92%
Proline	1.37%	15.68%	14.08%	2.09%
Methionine	1.35%	0.86%	1.02%	1.19%
Ornithine	1.03%	1.33%	1.61%	0.98%
1-Methylhistidine	0.60%	10.85%	17.19%	6.52%
threonine	0.54%	0.00%	0.34%	0.43%
Alanine	0.49%	0.20%	0.10%	0.34%
plusallo-σ-Hydroxylysine	0.47%	0.31%	0.15%	0.26%
Tyrosine	0.44%	0.34%	0.10%	0.13%
Glutamine	0.17%	0.27%	0.04%	0.08%
Asparagine	0.17%	0.69%	0.16%	0.33%
Arginine	0.15%	1.14%	0.57%	0.00%
alpha-Aminoadipic acid	0.12%	0.25%	0.09%	0.27%
o-Phosphoserine	0.11%	0.10%	0.13%	0.00%
Theanine	0.09%	0.55%	0.23%	0.00%

hydroxy-L-proline	0.00%	0.22%	0.19%	0.00%
Cystathionine	0.00%	0.89%	0.73%	0.45%
Histidine	0.00%	0.13%	0.13%	0.00%

Table S2

Organic acids	LLS	HX	ZLL	QM
Oxalic acid	3.40%	0.08%	0.25%	0.61%
Tartaric acid	0.78%	0.67%	0.63%	1.79%
Pyruvic acid	1.19%	1.36%	1.15%	1.54%
Malic acid	0.13%	0.00%	0.27%	0.02%
Lactic acid	0.42%	0.62%	0.20%	2.56%
Acetic acid	0.00%	0.00%	0.02%	0.01%
Pyroglutamic acid	16.14%	39.95%	39.08%	27.65%
Citric acid	0.11%	1.01%	0.03%	0.03%
Butanedioic acid	77.53%	56.18%	58.29%	65.48%
Fumaric acid	0.04%	0.04%	0.01%	0.01%
Ascorbic acid	0.25%	0.08%	0.07%	0.30%

Table S3

Count	Compound	CAS#	Formula	MW	RI	Rt [sec]	Dt [RIPrel]	Comment
1	Linalool oxide	C60047178	C10H18O2	170.3	1085.5	772.427	1.2535	
2	(E)-2-Octenal	C2548870	C8H14O	126.2	1055.5	731.308	1.3272	
3	1,8-Cineole	C470826	C10H18O	154.3	1038.6	706.95	1.7186	dimer
4	2-Ethyl-1-hexanol	C104767	C8H18O	130.2	1022.4	682.458	1.4013	
5	Heptanol	C53535334	C7H16O	116.2	974	599.627	1.3991	
6	2-Hexanol	C626937	C6H14O	102.2	972	595.796	1.5631	dimer

7	3-Methyl valeric acid	C105431	C6H12O2	116.2	933	523.158	1.5999	
8	heptanal	C111717	C7H14O	114.2	930.2	518.015	1.3401	
9	Octanal	C124130	C8H16O	128.2	995.1	637.633	1.385	
10	3-Furanmethanol	C4412913	C5H6O2	98.1	981.4	613.206	1.1081	
11	Diethylene glycol dimethyl ether	C111966	C6H14O3	134.2	964.3	581.618	1.1565	monomer
12	2-Acetylthiazole	C24295032	C5H5NOS	127.2	1027.8	690.812	1.1138	
13	(E)-2-Hexenal	C6728263	C6H10O	98.1	861.2	408.61	1.1898	
14	5-Methyl-3-heptanone	C541855	C8H16O	128.2	933.4	523.883	1.2835	monomer
15	(E)-2-Heptenal	C18829555	C7H12O	112.2	950.3	555.41	1.2534	monomer
16	Diethylene glycol dimethyl ether	C111966	C6H14O3	134.2	947.1	549.261	1.1742	diner
17	2,6-dimethylpyrazine	C108509	C6H8N2	108.1	922.5	504.143	1.1334	
18	Octamethyltrisiloxane	C107517	C8H24O2Si3	236.5	859.2	406.009	1.5524	
19	isobutyl acetate	C110190	C6H12O2	116.2	756.6	293.048	1.6139	

20	2-Methyl-1-pentanol	C105306	C6H14O	102.2	819.4	358.238	1.3039	
21	Methyl isovalerate	C556241	C6H12O2	116.2	793.7	330.593	1.2057	
22	Isopentan-1-ol	C123513	C5H12O	88.1	757.2	293.638	1.2406	
23	Pentanal	C110623	C5H10O	86.1	735.9	273.357	1.4323	dimer
24	Pyrazine, 2,5-dimethyl-	C123320	C6H8N2	108.1	904.5	473.056	1.5047	
25	2-Furanmethanol	C98000	C5H6O2	98.1	853.3	398.472	1.3899	dimer
26	2-ethylfuran	C3208160	C6H8O	96.1	737.8	275.184	1.305	
27	2-methylbutanol	C137326	C5H12O	88.1	735.7	273.169	1.4768	
28	1-pentanol	C71410	C5H12O	88.1	775.7	312.14	1.51	dimer
29	Pentanol	C71410	C5H12O	88.1	778.5	315.003	1.2514	monomer
30	Butyl propanoate	C590012	C7H14O2	130.2	883.5	439.824	1.2833	
31	Acetic acid butyl ester	C123864	C6H12O2	116.2	819.3	358.118	1.6142	dimer
32	Acetic acid butyl ester	C123864	C6H12O2	116.2	819.2	358.05	1.2342	monomer
33	ethyl-2-methyl propanoate	C97621	C6H12O2	116.2	722.2	261.372	1.5566	dimer
34	ethyl-2-methyl propanoate	C97621	C6H12O2	116.2	729.2	267.413	1.1979	monomer

35	Pentanal	C110623	C5H10O	86.1	665.2	221.456	1.4176	monomer
36	2-Ethoxybutane	C2679870	C6H14O	102.2	623.7	201.273	1.4489	
37	1-propanol	C71238	C3H8O	60.1	561.3	175.569	1.2456	
38	3-Pentanone	C96220	C5H10O	86.1	692.5	238.387	1.1	monomer
39	3-Pentanone	C96220	C5H10O	86.1	709.8	251.178	1.3496	dimer
40	2-Propanol	C67630	C3H8O	60.1	481.9	143.3	1.192	
41	Ethanol	C64175	C2H6O	46.1	440.3	126.395	1.1372	
42	Propionic acid	C79094	C3H6O2	74.1	743.7	280.654	1.1117	
43	1,2-Propanediol	C57556	C3H8O2	76.1	729.7	267.875	1.2689	
44	methyl butyrate	C623427	C5H10O2	102.1	742.3	279.357	1.1544	
45	2-methylpropanoic acid	C79312	C4H8O2	88.1	767.1	303.432	1.1531	monomer
46	2-methylpropanoic acid	C79312	C4H8O2	88.1	776.8	313.247	1.3724	dimer
47	2-furan methanol	C98000	C5H6O2	98.1	806.7	344.392	1.1334	monomer
48	(Z)-3-hexen-1-ol	C928961	C6H12O	100.2	876.8	430.075	1.2306	
49	ethyl propanoate	C105373	C5H10O2	102.1	678.3	229.094	1.1408	
50	acetoin (3-hydroxy-2-butanone	C513860	C4H8O2	88.1	748.9	285.603	1.3409	
51	Furfural	C98011	C5H4O2	96.1	792.3	329.22	1.3384	

52	2,3-Butanediol	C513859	C4H10O2	90.1	821.8	360.974	1.3616	
53	Triethylamine	C121448	C6H15N	101.2	671	224.716	1.2193	monomer
54	1-butanol	C71363	C4H10O	74.1	665.5	221.642	1.384	
55	2-Methylbutanal	C96173	C5H10O	86.1	625.2	201.924	1.3927	
56	2-Heptanone	C110430	C7H14O	114.2	844.4	387.398	1.2641	
57	2-cyclohexen-1-one	C930687	C6H8O	96.1	911.5	484.901	1.4037	
58	Methyl salicylate	C119368	C8H8O3	152.1	1150.8	860.157	1.2059	
59	hexanoic acid	C142621	C6H12O2	116.2	1022.9	683.252	1.3021	
60	Phenylacetaldehyde	C122781	C8H8O	120.2	1020.8	680.001	1.5399	dimer
61	benzaldehyde	C100527	C7H6O	106.1	997.1	641.113	1.4789	dimer
62	?alpha?-Pinene	C80568	C10H16	136.2	975.1	601.706	1.2214	monomer
63	Phenol	C108952	C6H6O	94.1	986.8	622.978	1.0747	
64	2-methyl-3-(methylthio)furan	C63012975	C6H8OS	128.2	967.7	588.003	1.1135	
65	Methylpyrazine	C109080	C5H6N2	94.1	824.2	363.675	1.0767	
66	N,N-diethylethanamine	C121448	C6H15N	101.2	690.5	236.986	1.4722	dimer
67	Propyl butanoate	C105668	C7H14O2	130.2	903.4	471.069	1.6883	
68	5-Methyl-3-	C541855	C8H16O	128.2	934.9	526.631	1.688	dimer

	heptanone							
69	Octanol	C111875	C8H18O	130.2	1048.1	720.859	1.4613	
70	2-Methoxy-vinylphenol	C7786610	C9H10O2	150.2	1338.2	1111.775	1.2138	
71	2-Octanone	C111137	C8H16O	128.2	974	599.668	1.3418	
72	Eugenol	C97530	C10H12O2	164.2	1341.1	1115.657	1.2883	
73	2,4-Heptadienal	C5910850	C7H10O	110.2	962.2	577.701	1.2033	
74	?alpha?-pinene	C80568	C10H16	136.2	954.3	562.835	1.6864	polymer
75	trans-2-Heptenal	C18829555	C7H12O	112.2	974	599.63	1.6696	
76	Propyl disulfide	C629196	C6H14S2	150.3	1126.8	827.885	1.2523	
77	Benzaldehyde	C100527	C7H6O	106.1	983.8	617.521	1.1439	monomer
78	3-Octanol	C589980	C8H18O	130.2	985.4	620.37	1.4032	
79	?alpha?-Pinene	C80568	C10H16	136.2	977.8	606.519	1.736	polymer
80	Allylacetic acid	C591800	C5H8O2	100.1	905.4	474.567	1.4358	
81	(E)-2-Pentenal	C1576870	C5H8O	84.1	770.6	306.893	1.1087	
82	butanal	C123728	C4H8O	72.1	598	190.478	1.0889	
83	3-Butenenitrile	C109751	C4H5N	67.1	638.3	207.83	1.1337	
84	phenylacetaldehyde	C122781	C8H8O	120.2	1041.7	711.552	1.256	monomer
85	1,8-Cineole	C470826	C10H18O	154.3	1045.4	716.977	1.2857	monomer
86	Limonene	C138863	C10H16	136.2	1038.2	706.456	1.2899	

87	2-Hexanol	C626937	C6H14O	102.2	977.4	605.883	1.2825	monomer
88	1-octen-3-one	C4312996	C8H14O	126.2	973.4	598.536	1.2783	
